# Rate of speech decline in individuals with amyotrophic lateral sclerosis

**DOI:** 10.1038/s41598-022-19651-1

**Published:** 2022-09-20

**Authors:** Marziye Eshghi, Yana Yunusova, Kathryn P. Connaghan, Bridget J. Perry, Marc F. Maffei, James D. Berry, Lorne Zinman, Sanjay Kalra, Lawrence Korngut, Angela Genge, Annie Dionne, Jordan R. Green

**Affiliations:** 1 Depatment of Communication Sciences and Disorders, MGH Institute of Health Professions, Boston, MA USA; 2 Department of Radiology, The Athinoula A. Martinos Center for Biomedical Images, MGH, Harvard Medical School, Boston, MA USA; 3 Department of Speech-Language Pathology, Rehabilitation Sciences Institute, University of Toronto, Toronto, ON Canada; 4 Hurvitz Brain Sciences Program, Sunnybrook Research Institute, Toronto, ON Canada; 5 KITE –Toronto Rehabilitation Institute – University Health Network, Toronto, ON Canada; 6 Department of Neurology, Sean M. Healey and AMG Center for ALS, MGH, Boston, USA; 7 Division of Neurology, Department of Medicine, University of Toronto, Toronto, ON Canada; 8 Sunnybrook Health Sciences Centre, Toronto, Canada; 9 Neuroscience and Mental Health Institute, University of Alberta, Edmonton, Canada; 10 Division of Neurology, University of Alberta, Edmonton, Canada; 11 Department of Clinical Neurosciences, Hotchkiss Brain Institute, University of Calgary, Calgary, Canada; 12 Department of Neurology and Neurosurgery, Clinical Research Unit, Montreal Neurological Institute & Hospital, Montreal, Canada; 13 Centre de Recherche du CHU de Québec - Université Laval, Montreal, Canada; 14 Program in Speech and Hearing Bioscience and Technology, Harvard University, Boston, USA

**Keywords:** Health care, Health occupations, Medical research, Neurology

## Abstract

Although speech declines rapidly in some individuals with amyotrophic lateral sclerosis (ALS), longitudinal changes in speech have rarely been characterized. The study objectives were to model the rate of decline in speaking rate and speech intelligibility as a function of disease onset site, sex, and age at onset in 166 individuals with ALS; and estimate time to speech loss from symptom onset. We also examined the association between clinical (speaking rate/intelligibility) measures and patient-reported measures of ALS progression (ALSFRS-R). Speech measures declined faster in the bulbar-onset group than in the spinal-onset group. The rate of decline was not significantly affected by sex and age. Functional speech was still maintained at 60 months since disease onset for most patients with spinal onset. However, the time to speech loss was 23 months based on speaking rate < 120 (w/m) and 32 months based on speech intelligibility < 85% in individuals with ALS-bulbar onset. Speech measures were more responsive to functional decline than were the patient-reported measures. The findings of this study will inform future work directed toward improving speech prognosis in ALS, which is critical for determining the appropriate timing of interventions, providing appropriate counseling for patients, and evaluating functional changes during clinical trials.

## Introduction

Most individuals with amyotrophic lateral sclerosis (ALS) progressively lose the ability to speak and, therefore, eventually require assistive devices to fulfill communication needs^1–4^. The onset and progression of dysarthria, the speech disorder associated with ALS, is a major concern of patients who invariably want to know “when” and “how fast” their loss of speech will occur. Despite the critical importance of accurate speech prognosis to clinical care, the longitudinal decline of speech has rarely been characterized, leading clinicians to speculate based on their selective and subjective experiences. Improving predictive models of speech decline is not only needed to better advise patients regarding the expected course of their disease, but also critical for optimizing ALS clinical trials and treatment strategies for maintaining communication abilities throughout the disease.

Although the course of overall functional decline due to ALS has been modeled extensively using clinical scales such as the Amyotrophic Lateral Sclerosis Functional Rating Scale-Revised (ALSFRS-R)^5–9^, the focus on speech decline has been limited. Studies of speech are needed because the rate of speech decline may be decoupled from that occurring in other parts of the motor system^5^ and the disease progression and expression are known to be very heterogeneous^5–13^. The few existing studies of longitudinal speech decline in ALS suggest that speech remains functional for an average of 18 months from the first bulbar symptom^14^. For individuals who experience their first symptom in bulbar muscle (i.e., bulbar-onset), speech tends to decline faster than for those with spinal-onset ALS^14^ and can decline as fast as 20% a year^15^. While these estimates are useful, they are derived from only a few studies, some of which had design issues that limit interpretation, including a relatively small number of participants. For instance, the estimates of speech decline in Makkonen et al.^14^ were based on only 9 participants (four bulbar and five spinal) who completed their follow-up session. In addition, the model of speech intelligibility decline provided by Rong et al.^15^ did not account for site of onset and was calculated in months from ALS diagnosis, which may be problematic as participants could be at different stages of their disease at the time of diagnosis. Ideally, predictive models of ALS speech decline will account for the influence of potential risk factors such as site of disease onset, age at onset, and sex on group and individual trends. Additional research is also needed to identify the most responsive markers of speech decline.

In the current study, we used measures of speaking rate and speech intelligibility as indices of speech function because of their widespread use in clinical evaluation of bulbar function, disease severity, and motor speech impairment in ALS. Bulbar involvement is often characterized by slowed speaking rate and reduced speech intelligibility^1,16^. During the course of speech decline, it has been reported that (1) changes in speaking rate precedes the onset of intelligibility decline in individuals with ALS, and (2) intelligibility declines in a faster rate when speaking rate passes the threshed of 120 w/m^1,17^. Further, speaking rate is significantly associated with clinical measures of overall ALS and bulbar disease severity, and has necessary psychometric properties (high reliability, construct validity, and sensitivity (AUC = 0.81)) to detect bulbar signs prior to the onset of overt bulbar symptoms (i.e., the presymptomatic stage)^18^. The aims of the study were to (1) identify the status of speaking rate and speech intelligibility in ALS subgroups at baseline relative to normative data accounting for sex and age; (2) determine the rate of decline in functional speech measures, while controlling for ALS site of onset, age at onset, and sex; (3) estimate the time to speech loss in ALS subgroups; (4) compare the slope of change in speech measures to the slope of change in self-reported ratings (the ALSFRS-R total score and bulbar subscore); and (5) determine if the decline in speech measures are associated with the decline in the ALSFRS-R total score and bulbar subscore. The comparative analysis between speech measures and ALSFRS-R scores is important because ALSFRS-R scores are commonly used in routine clinical evaluations, clinical trials, and patient-oriented research to estimate the overall progression of ALS. Results from this work may help clinicians improve estimates of the rate of speech decline and, as such, optimize patient care and the design of individualized treatment plans.

## Methods

### Participants

One hundred sixty-six individuals with ALS (71 females) aged from 33 to 86 years old (*M* = 58 years, *SD* = 10) were included in this study. All participants in the ALS group were diagnosed with ALS by a neurologist using the revised El Escorial criteria^19^ and had no history of other neurological disorders. The ALS group was subdivided by site of symptom onset. Thirty participants (18 females) exhibited bulbar onset, while 136 participants (53 females) exhibited spinal onset. Unequal sample sizes in the ALS-Spinal and ALS-bulbar groups are expectable given the higher prevalence of ALS-spinal compared to ALS-bulbar onset. It’s been reported that the prevalence of ALS patients being characterized as spinal onset predominates that of bulbar onset by almost 58–82%^20^. In addition, 167 healthy controls (HC) (76 males, 91 females) aged from 29 to 77 years old (*M* = 58 years, *SD* = 10 years) were included as controls. Control group participants had no history of any known neurological, cognitive, speech, or swallowing impairment. Healthy controls volunteered to participate in the study and were recruited through flyers and Ads.

The data obtained from individuals with ALS and healthy controls were collected from 2009 to 2018 at multiple sites in the United States and Canada to be a good representative of the population in North America. The study sites located in the United States included the MGH Institute of Health Professions (Boston), University of Nebraska, and University of Dallas. The study sites located in Canada included the Sunnybrook Research Institute in Toronto and the sites involved in the Canadian ALS Neuroimaging Consortium (CALSNIC)^21^. The number of participants recruited from the United States and Canada are provided in Table 1. All participants were native speakers of English and none had any reported hearing or vision impairment or literacy disability that could have impacted reading of speech stimuli. All participants with ALS as well as normal controls had no cognitive impairment as determined by the MoCA test. In addition, none of the subjects were on medications known to affect speech production. Written informed consent was obtained from all participants and the study was approved by the Institutional Review Board of all institutions involved in the data collection for this study. The study was conducted in accordance with the relevant guidelines and regulations outlined in the Declaration of Helsinki. Table 1 represents demographic and clinical information about the participants.

**Table 1:** Demographic and clinical features of participants.

	*n*	ALS Bulbar (*n* = 30)	*n*	ALS Spinal (*n* = 136)	*n*	ALS All (*n* = 166)	*n*	Healthy controls (*n* = 167)
**Site of study**								
United States		23		66		89		34
Canada		7		70		77		133
**Sex**								
Male		12		83		95		76
Female		18		53		71		91
**Age at baseline (years)**								
Mean (SD)	30	60.23 (11.12)	135	57.86 (9.84)	165	58.29 (10.09)	158	57.75 (9.91)
Range		40–86		33–82		33–86		29–77
**Time between sessions (months)**								
Mean (SD)	30	7.13 (6.41)	135	9.33 (7.01)	165	8.93 (6.94)		–
Range		1–27		1–36		1–36		–
**Time from symptom onset to baseline (months)**								
Mean (SD)	30	19.63 (12.14)	136	24.85 (14.49)	166	23.90 (14.21)		–
Range		3–50		1–58		1–58		–
**Time from diagnosis to baseline (months)**								
Mean (SD)	30	5.43 (6.02)	134	10.84 (10.33)	164	9.85 (9.89)		–
Range		0–26		0–56		0–56		–
**ALSFRS-R (session 1)**								
Total score mean (SD)	25	40.52 (3.89)	119	37.97 (6.13)	144	38.42 (5.87)		–
Total score range		32–46		21–48		21–48		–
Bulbar subscore mean (SD)	25	8.6 (1.58)	118	11.08 (1.40)	143	10.64 (1.71)		–
Bulbar subscore range		5–12		6–12		5–12		–
**ALSFRS-R (session 2)**								
Total score mean (SD)	26	35.62 (7.39)	113	31.36 (7.94)	139	32.16 (7.99)		–
Total score range		17–46		7–47		7–47		–
Bulbar subscore mean (SD)	26	7.04 (2.60)	113	10.27 (2.13)	139	9.66 (2.55)		–
Bulbar subscore range		2–11		2–12		2–12		–
**Speaking Rate (Session 1) (words per minute)**								
Mean (SD)	30	118.67 (34.62)	135	168.54 (33.32)	165	159.47 (38.62)	166	187.56 (28.08)
Range		55–176		82–256		55–256		119–253
**Speaking Rate (Session 2) (words per minute)**								
Mean (SD)	30	84.33 (36.51)	135	151.04 (44.63)	165	138.92 (50.29)		–
Range		32–170		19–258		19–258		–
**Intelligibility (Session 1) (% words intelligible)**								
Mean (SD)	27	92.93 (6.12)	136	97.77 (2.77)	163	96.96 (3.95)	166	98.42 (1.95)
Range		79–100		87–100		79–100		89–100
**Intelligibility (Session 2) (% words intelligible)**								
Mean (SD)	26	57.46 (33.44)	136	92.18 (17.71)	162	86.61 (24.50)		–
Range		0–100		2–100		0–100		–

Not all subjects had a data point for every statistic. Total sample sizes are reported in parentheses under each group name. The number of subjects, mean, SD, and range values are provided for each statistic.

*ALS* amyotrophic lateral sclerosis, *ALS-FRS-R* Revised Amyotrophic Lateral Sclerosis Functional Rating Scale, *SD* standard deviation.

### Procedure

Participants were seen across multiple sessions during which they recorded a variety of speech and non-speech tasks. There was a large variation across participants in terms of the number of data collection sessions (M = 3.5 sessions, SD = 3.82, min = 1 session, max = 25 sessions). In this heterogeneous dataset, a substantial portion of the participants had only two data points. Because creating linear models that have many data points for some individuals and only two data points for a large portion of individuals introduces model complexities and leads to overfitting, we analyzed changes between the first and last recordings of the Sentence Intelligibility Test (SIT)^22^ by each participant to obtain more accurate estimations of the rate of decline in speech measures. In fact, the change score is often used in clinical trial settings. For example, the combined assessment of function and survival (CAFS) score is a well-accepted method of analyzing clinical trial data that looks at the change between the first and last measurements^23^. Therefore, all the 166 participants with ALS had both the baseline and follow-up data and, there were no dropouts due to death or loss to follow-up. The time interval between the two sessions ranged from 1 to 37 months (M = 9.63, SD = 7.25).

### Functional speech measures

Measures of speaking rate and speech intelligibility were extracted from recordings of each participant’s reading of 11 randomly generated sentences from the SIT^22^. The number of words in each sentence ranged from five to 15. Participants were asked to read the sentences using their habitual speaking rate and loudness. Audio recordings of the participants were collected using the procedure reported in previous studies^11,15^. Subsequently, a trained research assistant who was unfamiliar with the SIT sentences and blind to the participants' diagnoses, listened to the audio recordings and transcribed the sentences produced by the participants. Speaking rate was calculated for each sentence as the number of words per minute (w/m) averaged across the 11 sentences. A percent intelligibility score for each sentence was calculated as the percent of intelligible (correctly transcribed) words out of the total number of words contained in the sentence. The percent intelligibility scores of all 11 sentences were averaged to derive the total percent intelligibility score. The inter and intrarater reliability of SIT transcriptions were reported in a recent study by our lab, which used a subset of data used in this study to create an empirical classification system for speech severity in patients with dysarthria secondary to ALS. Overall, the intraclass correlation (ICC) for intrarater transcription reliability was reported as 0.91 (p < 0.001) and the ICC for interrater transcription reliability ranged from 0.92 to 0.96 (p < 0.001)^24^, demonstrating excellent reliability in SIT transcription.

### Patient-reported measure of function

The ALSFRS-R is a well-established and reliable measure of global motor (i.e., bulbar, fine motor, gross motor, and respiratory) disability in patients with ALS^25^. It is commonly used in clinics to assess ALS disease progression and as an endpoint in ALS clinical trials and survival outcomes^7,26–28^. A measure of patient-reported bulbar function was derived from the ALSFRS-R bulbar subscore, which includes three items: speech, swallowing, and saliva management, with possible scores ranging from 0 (maximum disability) to 12 (normal function). Please refer to Table 1 for information about the ALSFRS-R total score and ALSFRS-R bulbar subscore of individuals with ALS at baseline and follow-up sessions.

### Statistical analyses

#### Examining the distribution of sex and age across groups

ANOVA test was used to examine whether there were any differences between the groups (HC, ALS-spinal, and ALS-bulabr) in terms of the age of participants. In addition, the proportion of male and female participants in each group was examined using chi-squared tests.

#### Statistical analyses related to the aims of the study

**Figure 1:**
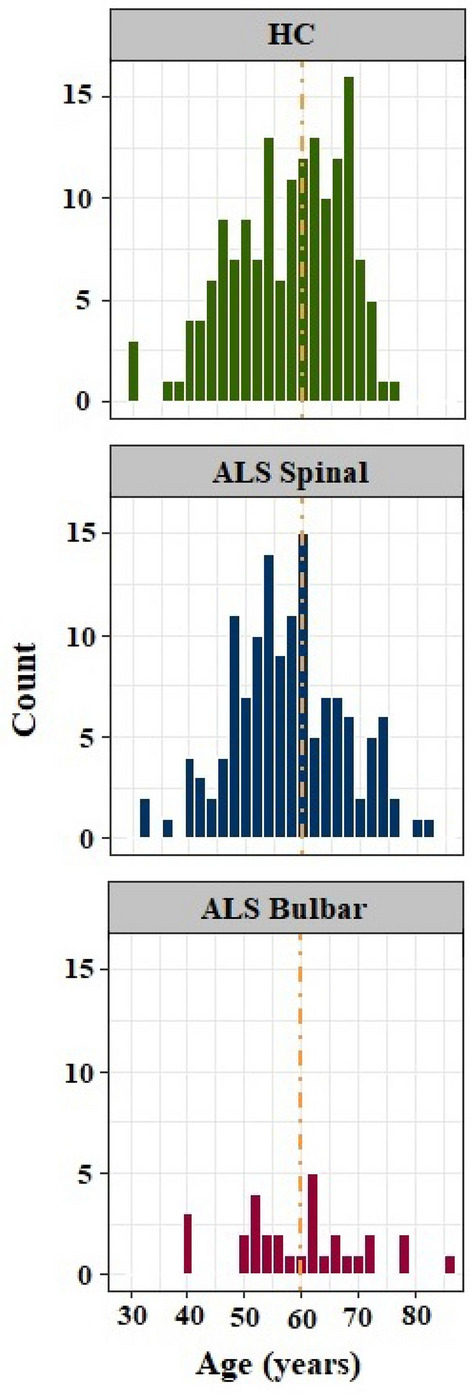
Age distribution in HC, ALS-spinal, and ALS-bulabar groups.

Aim 1 examined the ALS subgroups’ speaking rate and speech intelligibility at baseline relative to normative data accounting for sex and age. This aim facilitates interpretation of the longitudinal results by showing, where the individuals with ALS stand in terms of speech functions at baseline relative to normative data and how their speech change at various stages of the disease. Separate multiple regression analyses were used to examine the effect of group, sex, and age on speaking rate and speech intelligibility. In the regression models, the variable group had three levels [HC, ALS-spinal, and ALS-bulbar], and the variables sex, and age were binary and defined in two levels of analysis as follows: sex [males vs. females]; age at onset [younger than 60 years vs. older than 61 years]. The variable “age” was dichotomized because the cut-off point of 60 years yielded relatively equal sample sizes across the groups (Fig. 1). Subsequently, t-tests were performed to identify between-group differences. Bonferroni correction was used to control the family-wise error rate in multiple comparisons.

Aim 2 determined the rate of decline of functional speech measures while accounting for clinical and demographic factors. To address this aim, the lme() function from the nlme packages in R statistical software (i386 3.6.1), was used to fit linear mixed effects (LMEs) models on longitudinal speaking rate and intelligibility data. LMEs models were used to mitigate the effect of unequal time-interval between the baseline and follow-up sessions, as well as missing and unbalanced data, on the reliability of slope of change in the outcome measures^29,30^. In the first step, separate LMEs models were created for speaking rate and intelligibility with variables “time”, “site of onset”, “sex”, “age”, and (site of onset + sex + age) × time as fixed effects and “subject” as the random effect. In the next step, fixed effect variables that were not statistically significant were removed from the LMEs models using a backward selection approach. Subsequently, a simplified LMEs model was produced for the effect of site of onset on the rate of decline in speaking rate and intelligibility irrespective of sex and age (non-significant variables). In LMEs models, the time scale was based on months relative to baseline.

Because the baseline and follow-up sessions occurred at different time points relevant to the onset of ALS symptoms across all participants, the time (in months) of baseline and follow-up sessions were subtracted from the baseline time (in month) to align the baseline data for all subjects to 0 month from symptom onset. For example, if the baseline and follow-up sessions occurred at 5 and 12 months after symptom onset, the time points were shifted to 0 and 7 months, respectively. This technique allowed us to estimate the net rate of speech decline per month in each ALS subgroup (spinal-onset vs. bulbar onset). In the LMEs models, the variables of onset site, sex, and age were dichotomous and defined in two levels of analysis as follows: site of onset [spinal vs. bulbar]; sex [males vs. females]; age at onset [younger than 60 years at symptom onset (34–60 years, M = 52.63, SD = 5.88) vs. older than 61 years at symptom onset (61–88 years, M = 69, SD = 6.27)].

Aim 3 sought to estimate the time to speech loss. Speech loss was defined as either speaking rate of below 120 (w/m) or speech intelligibility below 85%^1,15,31^. Kaplan–Meier test was used to estimate the likelihood of maintaining speech functions based on the time to the occurrence of speaking rate below 120 (w/m), and intelligibility below 85%. These thresholds were selected based on the literature suggesting that speaking rate is a significant predictor of the intelligibility decline^1,17^ such that the slowness of speaking rate to approximately 120 words per minute (w/m), marks the initiation of accelerated decline in speech intelligibility (below 85%). Kaplan–Meier tests were fitted on the follow-up data (not the baseline) to estimate the fraction of individuals with ALS who maintain speech function until the follow-up session. Because all participants used in the study completed their follow-up session, there were no missing values due to mortality in our Kaplan–Meier analyses. In survival analysis models, the time scale was based on months since symptom onset which has been shown to be the best timescale in longitudinal studies of ALS, rendering models with increased precision and the lowest AIC^14,32^.

Aim 4 examined the difference in slope of change between speech measures and self-reported measures of ALSFRS-R (total score and bulbar subscore). To address this goal, separate pairwise t-tests were used for individuals in ALS-bulbar and ALS-spinal subgroups to compare the slope of change in speaking rate and intelligibility to the slope of change in the ALSFRS-R total score and ALSFRS-R bulbar subscore. The slope of change in speech and ALSFRS-R measures were calculated by subtracting the measures obtained at follow-up from measures obtained at baseline divided by the time interval. The p-value was adjusted (Bonferroni correction in multiple comparisons.

Aim 5 evaluated associations between changes in instrumental measures of speech function (intelligibility, speaking rate) and self-report ratings (the ALSFRS-R total score and bulbar subscore) across disease progression. The associations between the slope of change in functional speech measures and the slope of change in the ALSFRS-R total and bulbar subscores were assessed using the Pearson correlation tests. Bonferroni correction was performed to adjust the p-value in multiple tests.

## Results

### Distribution of sex and age across groups

The variable age demonstrated normal distribution in all groups as displayed in Fig. 1. The average age was almost similar in the ALS subgroups As well as healthy controls (between 58 and 60 years). The ANOVA tests showed no statistically significant differences among the three groups with regard to age. In addition, results of the chi-squared tests demonstrated that the proportion of male and female participants was only statistically significantly different in the ALS-spinal group (p = 0.01) with 83 individuals being male and 53 individuals being female in this group.

### Results related to study aims

**Figure 2:**
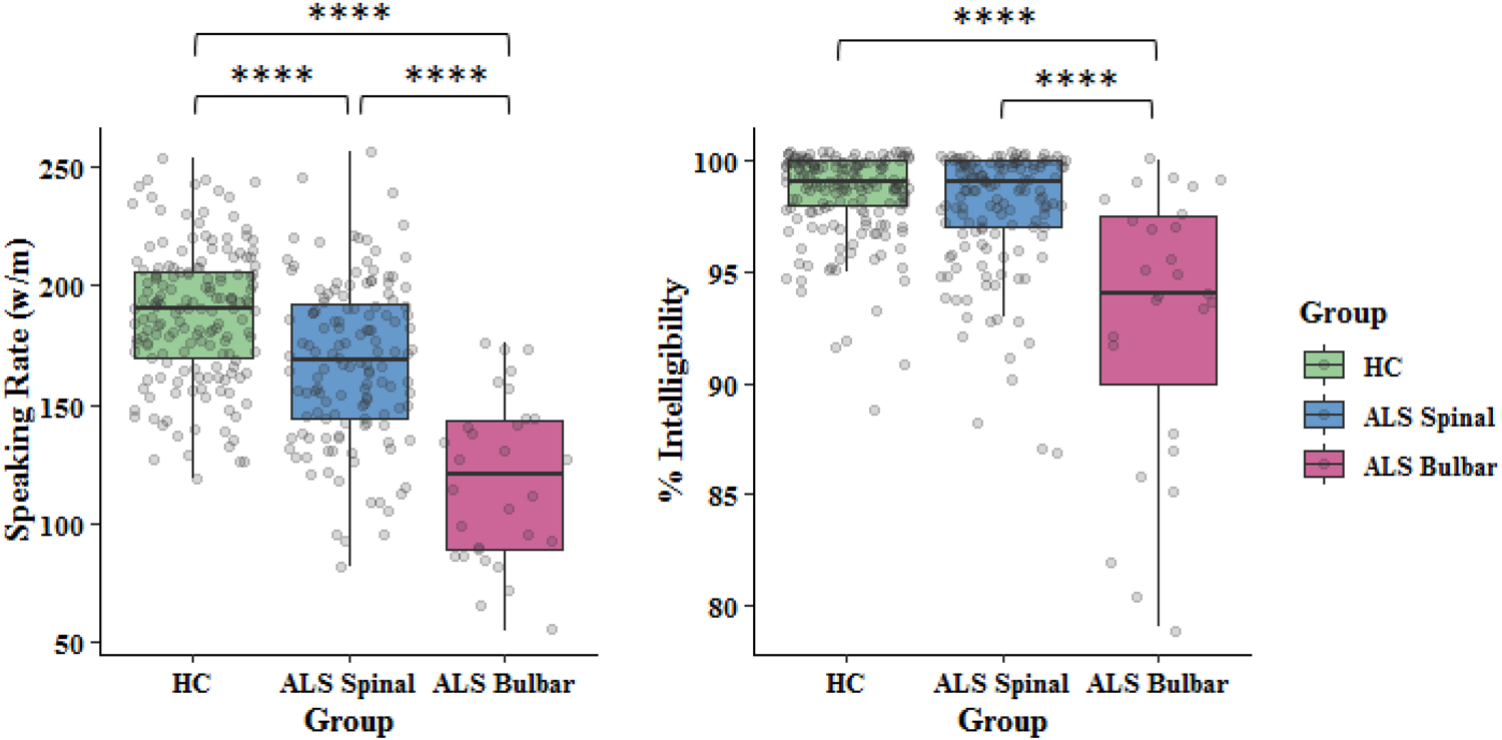
Baseline speaking rate and % intelligibility score in ALS-spinal, and ALS-bulbar groups relative to normative data (HC = healthy controls). ****Represents comparisons that remained significant after Bonferroni adjustment of p-values (p-values less than 0.017 was considered statistically significant).

Multiple regression analyses revealed significant effect of group on both speaking rate and intelligibility (Table 2). Neither sex nor age appeared to be significant factors in speaking rate differences observed between healthy controls and ALS subgroups. However, sex was observed to have a statistically significant effect on speech intelligibility such that females demonstrated a better speech intelligibility by only 0.78% (less than 1%) compared to men, which is negligible and not clinically meaningful. Mean (SD) values of baseline speaking rate (w/m) in healthy controls, ALS-spinal, and ALS-bulbar groups were 187.56 (28.08), 168.54 (33.32), and 118.67 (34.62), respectively. Mean (SD) values of baseline intelligibility scores in healthy controls, ALS-spinal, and ALS-bulbar groups were 98.42% (1.95), 97.76% (2.77), and 92.93% (6.12), respectively. T-tests indicated that speaking rate was significantly lower for the ALS-spinal (p < 0.001) and ALS-bulbar (p < 0.001) groups than for the HC group and for the ALS-bulbar group relative to the ALS-spinal group (p < 0.001). In addition, the baseline measure of intelligibility in the ALS-bulbar group was significantly lower than intelligibility in both the ALS-spinal and the HC groups (p < 0.001). The observed difference between the HC and ALS-spinal groups in speech intelligibility (p = 0.02) did not reach the adjusted p-value for multiple comparisons (p = 0.017) and was considered not significant (Fig. 2).

**Table 2:** Multiple regression analyses to estimates the effects of group, sex, and age on functional speech measures.

	Beta	SE	*P*-value
**Speaking rate**			
Intercept (HC)	202.98	7.70	< 0.00001
ALS-Spinal	− 21.64	3.65	< 0.00001
ALS-Bulbar	− 69.68	6.11	< 0.00001
Sex (Female)	− 4.48	3.47	0.197
Age at onset (Older)	− 4.97	3.46	0.152
**Intelligibility**			
Intercept (HC)	97.83	0.72	< 0.00001
ALS-Spinal	− 0.50	0.34	0.141
ALS-Bulbar	− 5.48	0.59	< 0.00001
Sex (Female)	0.81	0.32	0.013
Age at onset (Older)	− 0.48	0.32	0.139

**Figure 3:**
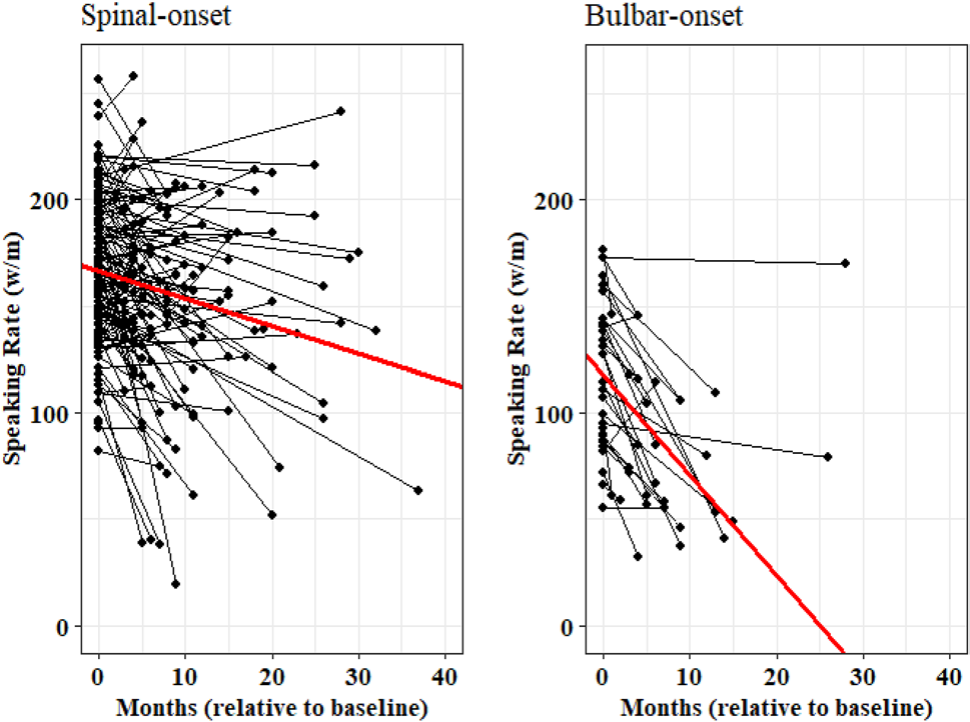
Speaking rate decline (w/m) in individuals with spinal-onset (left plot) and bulbar-onset (right plot) ALS.

**Figure 4:**
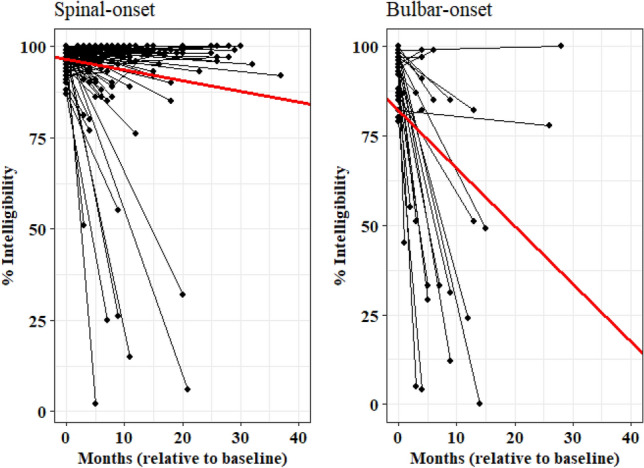
Speech intelligibility (%) decline in individuals with spinal-onset (left plot) and bulbar-onset (right plot) ALS.

Results of LMEs models for speaking rate and intelligibility demonstrated a significant effect of site of onset and a significant time × site of onset interaction (Table 3). Results of simplified LMEs models demonstrated that speaking rate is significantly lower in the ALS-bulbar group compared to ALS-spinal group by approximately 51 (w/m) at time 0 (baseline) and declines at a significantly faster rate (Table 4). While the slope of speaking rate declines by 1.74 (w/m) per month in individuals with spinal onset ALS, it declines by 4.16 (w/m) per month in individuals with bulbar onset ALS (Fig. 3). Similarly, speech intelligibility was significantly lower and showed faster rate of decline in the ALS-bulbar than ALS-spinal group. Individuals in the ALS-bulbar group demonstrated about 14% lower intelligibility at time 0 compared to those in the ALS-spinal group. The slope of decline in intelligibility of the ALS-bulbar group was steeper by about 1.3% per month compared to that of ALS-spinal group (1.61% vs. 0.28% decline per month in the intelligibility scores (words understood) of ALS-bulbar and ALS-spinal groups respectively) (Fig. 4).

**Table 3:** LMEs model estimates accounting for the effect of site of onset, sex, age at onset, and their interactions with time (i.e., months relative to baseline) on functional speech measures.

	Beta	SE	*P*-value
**Speaking rate**			
Intercept	169.17	4.72	< 0.00001
Time	− 1.07	0.33	0.0013
Site of onset (Bulbar)	− 52.10	7.74	< 0.00001
Sex (Female)	− 10.75	6.09	0.0796
Age at onset (Older)	1.85	5.95	0.7556
Time × site of onset (Bulbar)	− 1.22	0.65	0.0619
Time × sex (Female)	− 0.39	0.44	0.3704
Time × Age at onset (Older)	− 0.09	0.45	0.8294
**Intelligibility**			
Intercept	97.29	1.73	< 0.00001
Time	− 0.33	0.19	0.082
Site of onset (Bulbar)	− 13.99	2.96	< 0.00001
Sex (Female)	− 2.60	2.27	0.253
Age at onset (Older)	0.32	2.22	0.88
Time × site of onset (Bulbar)	− 1.36	0.39	0.0006
Time × sex (Female)	0.31	0.26	0.236
Time × Age at onset (Older)	− 0.20	0.27	0.457

**Table 4:** LMEs model estimates accounting for the effect of site of onset and its interaction with time (i.e., months relative to baseline) on functional speech measures.

	Beta	SE	*P*-value
**Speaking rate**			
Intercept (Spinal)	167.18	2.89	< 0.00001
Time	− 1.74	0.30	< 0.00001
Site of onset (Bulbar)	− 51.42	6.74	< 0.00001
Time × site of onset	− 2.42	0.79	0.0025
Speaking rate = 167.18 − 1.74 (months) − 51.42 (site of onset) − 2.42(months × site of onset)			
**Intelligibility**			
Intercept (Spinal)	96.38	1.2	< 0.00001
Time	− 0.28	0.14	< 0.041
Site of onset (Bulbar)	− 14.46	2.90	< 0.00001
Time × site of onset	− 1.33	0.38	0.0006
Intelligibility = 96.38 – 0.28 (months) – 14.46 (site of onset) – 1.33 (months × site of onset)

**Figure 5:**
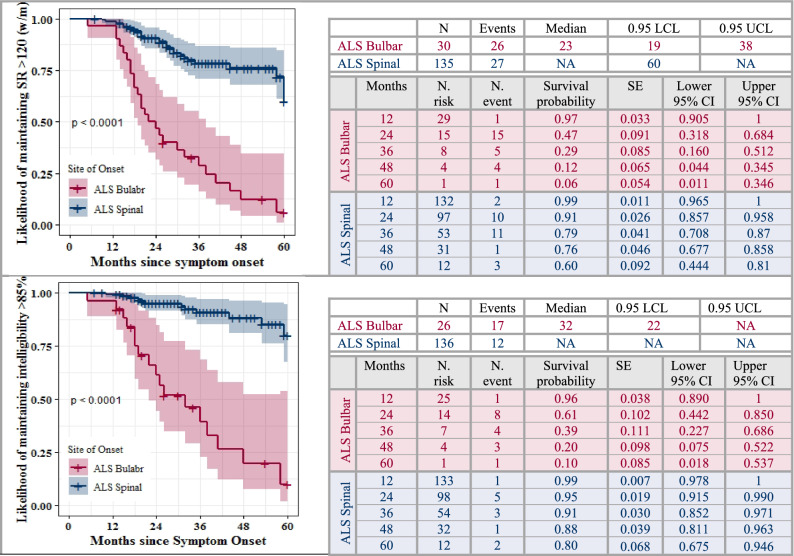
Kaplan–Meier curve and the corresponding estimates for time to speech loss defined as speaking rate below 120 (w/m) or intelligibility below 85%.

Kaplan–Meier tests demonstrated significantly reduced likelihood of maintaining speech functions at 12, 24, 36, 48, and 60 months since symptom onset for individuals with bulbar-onset compared to those with spinal onset (Fig. 5). While the median time to exhibiting a speaking rate lower than 120 (w/m) was 23 months for those with bulbar-onset ALS, 60% of participants with spinal-onset ALS maintained a speaking rate greater than 120 (w/m) over the 60-month follow-up.

Individuals in the ALS-spinal group were more resilient to intelligibility decline than speaking rate decline over the 60-month follow-up. About 80% of individuals in the ALS-spinal group maintained intelligibility greater than 85% during the 60-month follow-up period. However, the median time to exhibiting intelligibility lower than 85% was 32 months from symptom onset for the ALS-bulbar group.

In individuals with bulbar-onset ALS, the slope of change in both speaking rate and speech intelligibility were observed to be significantly faster than the slope of change in their ALSFRS total score and bulbar subscore. In individuals with spinal-onset ALS, however, the only significant difference in slope of change was between speaking rate and ALSFRS-R bulbar subscore, such that speaking rate declined faster than the ALSFRS-bulbar score in that group (Table 5).

**Table 5:** Mean (SD) of slope of change in speech measures and ALSFRS-R total score and ALSFRS-bulbar subscore in ALS bulbar and spinal groups.

	Descriptive Stat	t-test *P*-value (Cohen’s *d*)	
	Mean (SD)	ALSFRS-R Total score	ALSFRS-R Bulbar subscore
**ALS-Bulbar**			
Speaking rate	− 7.61 (7.92)	*P* = 0.001* (5.84)	*P* < 0.001* (5.66)
Intelligibility	− 9.43 (11.37)	*P* = 0.003* (8.07)	*P* = 0.001* (7.93)
ALSFRS-R total score	− 1.20 (2.27)		
ALSFRS-R Bulbar subscore	− 0.40 (0.66)		
**ALS-Spinal**			
Speaking rate	− 1.90 (5.15)	*P* = 0.279 (3.84)	*P* < 0.001* (3.59)
Intelligibility	− 0.80 (2.62)	*P* = 0.031 (2.30)	*P* = 0.018 (1.84)
ALSFRS-R total score	− 1.38 (2.07)		
ALSFRS-R Bulbar subscore	− 0.21 (0.41)		

T-test results for the comparison between longitudinal changes in speech measures and ALSFRS scores are also provided. Asterisks represent comparisons that remained statistically significant after adjusting p-values for multiple comparisons (adjusted p-value = 0.0125).

Finally, Pearson correlation tests indicated that only in the ALS-spinal group, there were statistically significant associations between the slope of decline in speech intelligibility and ALSFRS-R total score and ALSFRS-R bulbar subscore as well as between speaking rate and ALSFRS-R bulbar subscore. However, the observed correlations were mild and below *r* = 0.50 (Table 6).

**Table 6:** Pearson correlation coefficients for the association between longitudinal changes in speech measures and ALSFRS scores.

	Pearson coefficient (*P*-value)	
	ALSFRS-R total score	ALSFRS-R Bulbar subscore
**ALS-Bulbar**		
Speaking rate	− 0.16 (*P* = 0.455)	0.10 (*P* = 0.647)
Intelligibility	− 0.28 (*P* = 0.197)	− 0.38 (*P* = 0.075)
**ALS-Spinal**		
Speaking rate	0.20 (*P* = *0.031*)	0.31 (*P* < *0.001*) *
Intelligibility	0.31 (*P* < *0.001*) *	0.46 (*P* < *0.001*) *

Asterisks represent correlations that remained statistically significant after adjusting p-values for multiple testing (adjusted p-value = 0.0125).

## Discussion

Our findings indicate that individuals in the ALS-spinal and ALS-bulbar groups had slower rates of speech compared to normal controls at baseline. Baseline speech intelligibility of individuals with bulbar-onset ALS was also lower than normal controls. The findings also suggested that the rate of decline in speech measures were driven primarily by site of symptom onset rather than sex or age at onset. Group trends showed that on average, speaking rate and intelligibility scores were lower (by approximately 51 w/m and 14% words understood) in the bulbar-onset ALS group compared to the spinal-onset ALS group at baseline. In addition, the rates of decline in speaking rate and intelligibility were faster in the bulbar-onset ALS group (4.16 w/m per month, 1.61% words understood per month) than in the spinal-onset ALS group (1.74 w/m per month, 0.28% words understood per month). Although the rate of decline varied significantly across individuals, the median time to speech loss defined as speaking rate slower than 120 (w/m) or speech intelligibility below 85% was relatively short for those with bulbar-onset ALS. While the median time to exhibit a speaking rate slower than 120 (w/m) and ineligibility below 85% was respectively 23 and 32 for the bulbar-onset ALS group, functional speech was still maintained for the majority of individuals in the spinal-onset group over 60 months since symptom onset. In fact, at 60 months (5 years) after symptom onset, 60% of participants with spinal-onset ALS exhibited a speaking rate greater than 120 (w/m) and 80% of them exhibited speech intelligibility greater than 85%. Finally, clinical speech measures (i.e., speaking rate and speech intelligibility) were more responsive to functional decline than were the patient-reported measures (i.e., ALSFRS-R total score and bulbar subscore). The findings of this study have important implications for future work directed toward improving speech prognosis in ALS, which is critical for determining the appropriate timing of interventions, providing appropriate counseling for patients, and evaluating functional changes during clinical trials.

### ALS site of onset: the major influential factor for the rate of decline in speech functions

Literature on the effect of onset site on ALS progression has consistently reported that patients with bulbar-onset ALS demonstrate deterioration of functional measures (e.g., clinical scales) at a markedly increased rate compared to individuals with spinal-onset ALS. The majority of these studies used the ALSFRS-R^25^ as a prognostic index to assess global function in patients with ALS and its rate of decline^7,26–28,33^. Consistent with these self-reported measures, our models demonstrated significantly faster decline of speaking rate and intelligibility in individuals with bulbar-onset ALS. Additionally, our findings are consistent with previous findings of significant differences in speaking rate and/or intelligibility decline for those with bulbar vs. spinal-onset ALS^17,34^.

Our models also confirm prior work suggesting that speaking rate declines with a steeper slope than speech intelligibility over the course of the disease regardless of site of onset^1,17,35,36^. Speaking rate has been found to be a significant predictor of intelligibility decline, with the accelerated decline in speech intelligibility (below 85%) not occurring until speaking rate has slowed to approximately 120 w/m^1,17^.

Although several studies on models of ALS disease progression have attributed a faster rate of functional decline in older patients and females than in younger patients and males^32,37,38^, findings of this study suggested that rates of speech decline are driven primarily by site of symptom onset and are not influenced by sex or age. Our findings converge with Makkonen et al.^14^, who reported that among onset site, sex, and age, only the onset site significantly predicted the rate of decline in speech measures of individuals with ALS.

### Within-group variability in rate of speech decline was large for individuals with spinal and bulbar-onset ALS

The rate of speech decline for some individuals in both the spinal and bulbar groups diverged significantly from that of the overall group trend (e.g., faster rate of decline in some individuals in the ALS-spinal group and slower rate of decline in the ALS-bulbar group). Although the divergence only occurred in a small number of cases in each group, these findings provide further evidence in support of motor phenotype heterogeneity in ALS^10^ and exemplify the need for improved patient stratification. Therefore, objective criteria such as imaging-based biomarkers^39^, brain/plasma proteomics^40^, or assessment of bulbar motor involvement^13^ are necessary.

Results of this work suggest that speech measures can be used to uncover covert patient subgroups within the ALS-spinal and ALS-bulbar groups. Stratifying individuals with ALS into clinically relevant subgroups contributes to a better understanding of disease mechanisms and prognosis. In addition, subgroup analyses may enhance the quality of care for ALS patients by informing decision making, optimizing clinical trial planning/interpretation, and contributing to the development of effective therapeutic options.

### Time to speech loss in individuals with bulbar ALS is relatively short

Because ALS progresses rapidly in some affected individuals, an important goal of clinical management is to predict functional changes in patients’ speech performance in order to facilitate intervention planning, such as the introduction of assistive technology before the patient’s ability to learn these skills is hindered by the severity of their condition^2,41,42^. An important finding of this study was that the median time to speech loss based on speaking rate slower than 120 (w/m), was approximately 23 months for individuals with bulbar-onset ALS. In contrast, 60% of individuals with spinal-onset ALS were observed to maintain functional speech at 60 months since symptom onset. If we use speech intelligibility below 85% to index speech loss, the median time to speech loss was 32 months since symptom onset for individuals with bulbar-onset ALS. At 60 months since symptom onset, 80% of participants with spinal-onset ALS maintained speech intelligibility greater than 85%, whereas only 10% of individuals with bulbar-onset ALS demonstrated speech intelligibility greater than 85%. Rong et al.^15^ modeled time to speech loss in individuals with ALS and found that at 2.5 months after diagnosis, 71% of participants with ALS maintained sentence intelligibility scores higher than 85% and 41% of them demonstrated a speaking rate faster than 120 (w/m). At three years after diagnosis, however, only about 20% of their participants with ALS exhibited a speaking rate greater than 120 (w/m) and 30% of them had sentence intelligibility greater than 85%. Differences in study findings can likely be attributed to the fact that their model was based on time from diagnosis (years) rather than time since symptom onset. Considering time from diagnosis may be problematic because diagnostic criteria can vary across clinics and it may, therefore, capture different stages of disease across individuals with ALS. In addition, Rong et al.^15^ included individuals with ALS irrespective of the site of onset while clinical observations and empirical evidence have consistently indicated that the rate of disease progression and functional decline is significantly more rapid in individuals with bulbar onset than spinal onset^26,43,44^.

### Objective speech measures may be better markers of functional decline than patient-reported measures

Research has documented increased sensitivity and responsiveness of instrumental bulbar measures over patient-reported measures of bulbar dysfunction^45^. Our findings further support this assertion, as the slope of change in speech measures was statistically greater than the slope of change in ALS functional measures (i.e., ALSFRS-R total score and ALSFRS-R bulbar subscore). The slower rate of decline in clinical measures of ALS function may be expected as a result of the multidimensionality of the ALSFRS-R total score and the ALSFRS-R bulbar subscore, which is different from speech measures that objectively represent performance of participants in a single domain (i.e., speech). In addition, the change over time in speech measures was mildly associated with changes in ALS functional measures only for the spinal onset group. This finding is comparable with a study conducted by Barnett et al.^18^, who reported only mild-to-moderate associations between speaking rate and clinical measures of overall ALS and bulbar disease severity. These findings suggest that ALSFRS-R scores—including the bulbar subscore—do not effectively capture functional decline in speech motor performance over the course of bulbar decline, particularly in individuals with bulbar-onset ALS with greater speech impairment severity and a faster rate of decline in functional speech measures.

## Conclusions and study limitations

In sum, this study quantified the rate of decline in functional speech measures controlling for important clinical and demographic factors and provided estimates of time to speech loss in individuals with spinal and bulbar-onset ALS. These findings inform our understanding of speech decline over the course of ALS disease progression and will contribute to the literature supporting best practices for timely intervention. A number of limitations should, however, be acknowledged. First, because of the clinical heterogeneity of ALS, each patient may demonstrate a unique trajectory of speech decline, making prediction within an individual difficult. Although we accounted for factors such as onset site, age at onset, and sex, the models presented in this study only provided simplistic estimates of the rate of decline in functional speech measures of individuals with ALS. Multiple pathophysiological, clinical, epidemiological, and genetic factors singly or in combination, influence the rate of ALS progression. However, producing models of ALS progression that simultaneously account for these variables is almost impractical due to high inter-individual variability in the underlying pathophysiological and clinical manifestations of ALS, heterogeneity in the expression of the disease symptoms, collinearity among predictors, and complex interactions of multivariate factors. Second, the distribution of sex was unbalanced between the ALS-spinal and ALS-bulbar groups, which despite the robustness of LMEs models to unequal sample sizes, might have influenced the findings related to the effect of sex on speech measures of ALS subgroups. Third, we did not track the “time of the day” when the ALSFRS was scored and if that can influence the association between the ALSFRS scores and speech measures. Although we were not able to examine this, it has been reported that “time of day” does not significantly influence speech measures^46^. In addition, the data used in this study were collected during visits that took place during a relatively confined time window (between 10:00 am and 4:00 pm rather than early morning and late at night) which could minimize the potential confounding effect of “time of day” on speech measures.

## Data Availability

The datasets used and/or analysed during the current study will be made available from the corresponding author on reasonable request.
